# Association between temperature and mortality: a multi-city time series study in Sichuan Basin, southwest China

**DOI:** 10.1265/ehpm.23-00118

**Published:** 2024-01-12

**Authors:** Yizhang Xia, Chunli Shi, Yang Li, Shijuan Ruan, Xianyan Jiang, Wei Huang, Yu Chen, Xufang Gao, Rong Xue, Mingjiang Li, Hongying Sun, Xiaojuan Peng, Renqiang Xiang, Jianyu Chen, Li Zhang

**Affiliations:** 1 Sichuan Provincial Center for Disease Control and Prevention, No. 6, Zhongxue Road, Wuhou District, Chengdu 610041, China; 2 Zigong Center for Disease Control and Prevention, No. 826, Huichuan Road, Ziliujing District, Zigong 643000, China; 3 School of Public Health, Chengdu Medical College, No. 783, Xindu Road, Xindu District, Chengdu 610500, China; 4 Chengdu Center for Disease Control and Prevention, No. 6, Longxiang Road, Wuhou District, Chengdu 610041, China; 5 Guangyuan Center for Disease Control and Prevention, No. 996, Binhebei Road, Lizhou District, Guangyuan 628017, China; 6 Panzhi hua Center for Disease Control and Prevention, No. 996, Jichang Road, Dong District, Panzhi hua 617067, China; 7 Mianyang Center for Disease Control and Prevention, No. 50, Mianxingdong Road, Gaoxin District, Mianyang 621000, China; 8 Yaan Center for Disease Control and Prevention, No. 9, Fangcao Road, Yucheng District, Yaan 625000, China; 9 Fucheng Center for Disease Control and Prevention, No. 116, Changhong Road, Fucheng District, Mianyang 621000, China

**Keywords:** Ambient temperature, Non-accidental mortality, Attributable risk, Time series

## Abstract

**Background:**

There are few multi-city studies on the association between temperature and mortality in basin climates. This study was based on the Sichuan Basin in southwest China to assess the association of basin temperature with non-accidental mortality in the population and with the temperature-related mortality burden.

**Methods:**

Daily mortality data, meteorological and air pollution data were collected for four cities in the Sichuan Basin of southwest China. We used a two-stage time-series analysis to quantify the association between temperature and non-accidental mortality in each city, and a multivariate meta-analysis was performed to obtain the overall cumulative risk. The attributable fractions (AFs) were calculated to access the mortality burden attributable to non-optimal temperature. Additionally, we performed a stratified analyses by gender, age group, education level, and marital status.

**Results:**

A total of 751,930 non-accidental deaths were collected in our study. Overall, 10.16% of non-accidental deaths could be attributed to non-optimal temperatures. A majority of temperature-related non-accidental deaths were caused by low temperature, accounting for 9.10% (95% eCI: 5.50%, 12.19%), and heat effects accounted for only 1.06% (95% eCI: 0.76%, 1.33%). The mortality burden attributable to non-optimal temperatures was higher among those under 65 years old, females, those with a low education level, and those with an alternative marriage status.

**Conclusions:**

Our study suggested that a significant association between non-optimal temperature and non-accidental mortality. Those under 65 years old, females, and those with a low educational level or alternative marriage status had the highest attributable burden.

**Supplementary information:**

The online version contains supplementary material available at https://doi.org/10.1265/ehpm.23-00118.

## Background

Extreme weather and climate change continue to cause concern among researchers worldwide, and the increase in the frequency and intensity of extreme weather events poses a threat to human health [1–3]. Numerous studies in the past have confirmed the adverse effects of extreme temperatures on human health [4–8]. Recent studies have found the significant associations between non-optimal temperatures and increased risk of mortality of cardiovascular (CVD), nervous system and respiratory diseases [9–12]. Additionally, a study in China reported that non-optimal temperatures could rapidly increase the risk of acute heart attack [13]. More than five million people worldwide die each year from extreme heat or cold under a changing global climate, accounting for 9.43% of all deaths according to a 20 year cohort study [14]. Non-optimal temperature is a very important health hazard, and further research is needed to reveal its adverse effects on human health.

A wide range of multicenter studies is available in developed countries. Gasparrini et al. found that the burden of non-accidental deaths attributable to non-optimal temperatures was 5.86% and 6.52% in 135 cities in the United States and 51 cities in Spain, respectively [15]. In recent years, there has also been a growing number of multicenter studies on the effects of temperature on human health in China. For example, Chen et al. found that the attributable burden of cardiovascular disease mortality caused by non-optimal temperatures in 272 cities in China was 17.48% [16]. Effects of non-optimal temperatures on nervous system diseases were assessed in five Chinese cities [9]. The effects of non-optimal temperatures on human health were measured in 31 cities [17], 43 counties [18], and 66 communities [19] in China.

The Sichuan Basin is located in the south-central part of the Asian continent, in the heart of China. A study found that since 1960, the annual average temperature of the Sichuan Basin has increased at a rate of 0.17 °C per decade [20]. Some studies predict that by 2030, the Sichuan Basin will be about 1 °C warmer than the decadal average temperature observed in 2000. This warming will continue beyond 2060, and by the end of the century, the annual average temperature will have likely exceeded 20 °C [21]. In the past, most studies have analyzed the effects of single-city non-optimal temperatures on human health in the Sichuan Basin. For example, Cui et al. found that the attributable risks of respiratory and cardiovascular diseases caused by non-optimal temperatures in Chengdu in the Sichuan Basin were 19.69% and 11.40%, respectively, with cold responsible for a higher proportion of deaths than heat [22]. Yin et al. found an association between temperature and the incidence of HFMD. High temperatures have acute and short-term effects, while the effects of low temperatures will persist for longer periods of time. Males and children under one year of age were more vulnerable to temperature changes [23]. Therefore, a multicenter study based on previous single-city studies was conducted to more accurately reflect the effect of temperature on population health in southwest China.

We collected data from four cities in the Sichuan Basin, southwest China. We also investigated the impact of both high and low temperatures on the mortality risk of patients with non-accidental diseases, and examined the vulnerable populations by age, gender, education level, and marital status in a stratified analysis. The results of the study will provide valuable information for developing effective intervention measures and public health policies in the Sichuan Basin, southwest China.

## Methods

### Study area

**Fig. 1:**
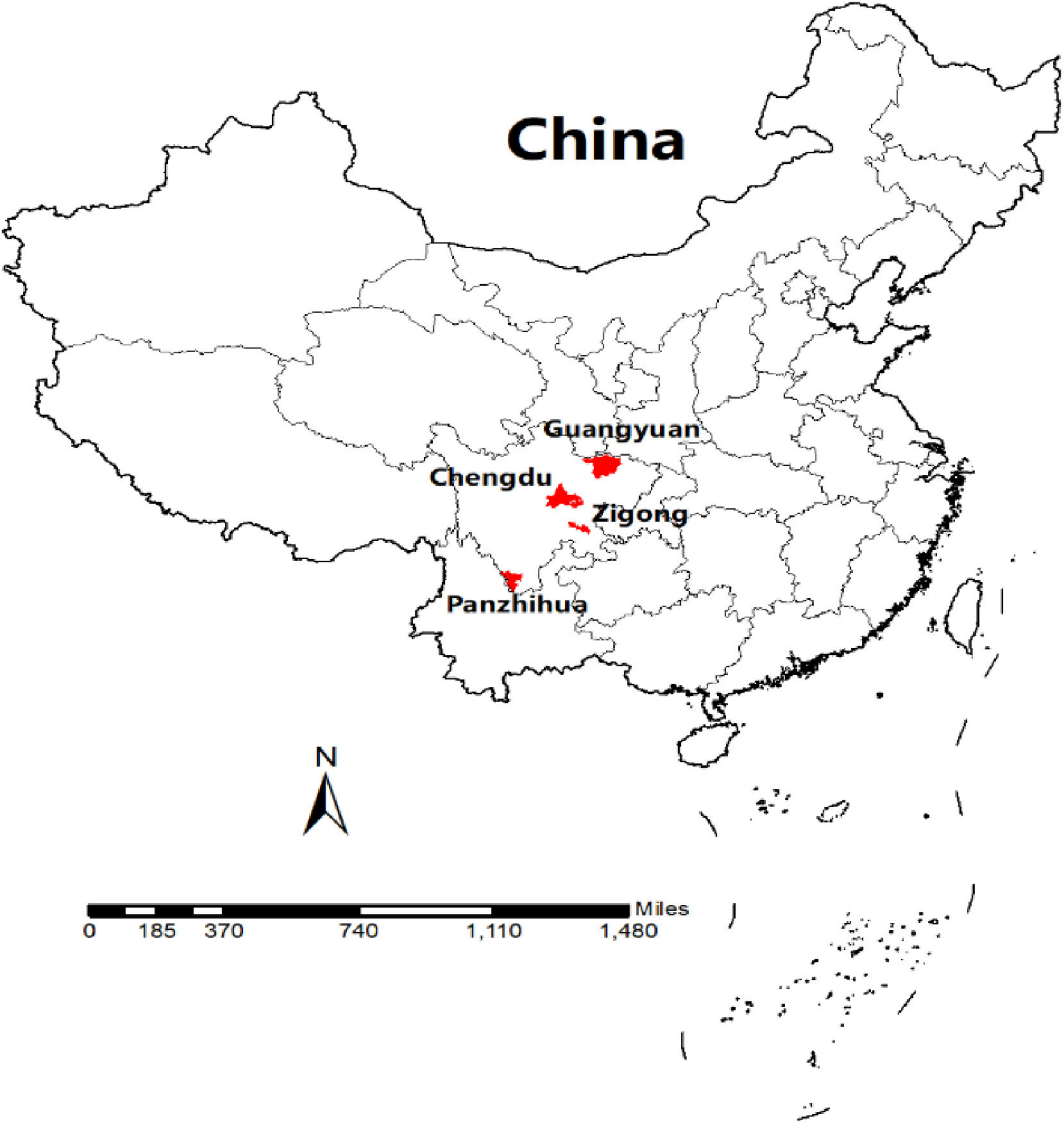
Geographical distribution of the studied cities.

The Sichuan Basin, located in southwest China, is one of the four major basins in China. The climate of the basin belongs to the humid sub-tropical monsoon type, with high temperatures in the east and low temperatures in the north and west. In addition, it is one of the most populous regions in China and in the world. The four study cities were Chengdu, Zigong, Guangyuan, and Panzhihua. Chengdu (east longitude: 102°54′–104°53′ and north latitude: 30°05′–31°26′) is located in the central part of the Sichuan Basin and is the provincial capital city of the Sichuan Basin. Zigong (east longitude: 102°02′–105°16′ and north latitude: 28°55′–29°38′) is located in the southern part of the Sichuan Basin. Guangyuan (east longitude: 104°36′–106°45′ and north latitude: 31°31′–32°56′) is located in the northern part of the Sichuan Basin. Panzhihua (east longitude: 101°08′–102°15′ and north latitude: 26°05′–27°21′) is located in the southern part of the Sichuan Basin (Figure 1).

### Mortality data

Daily non-accidental deaths for the four cities were obtained from the Population Death Information Registration Management System (PDIRMS), which covers all the mortality information of residents in the four cities. Deaths of residents were confirmed by hospitals or doctors in the residents’ homes, and the data were recorded in the system. Due to availability, the data covered different periods of time in the four study cities. Chengdu and Zigong had records from January 1, 2016, to December 31, 2021; Guangyuan and Panzhihua had records from January 1, 2018, to December 31, 2021. According to the International Classification of Diseases, Tenth Revision (ICD-10), A00-R99 is classified as non-accidental deaths (referred to as “total deaths” in this study; ICD-10: A00-R99). Finally, a stratified analysis was performed by gender, age group (0–64 years old and ≥65 years old), education level (low education or high education), and marital status (married or alternative marriage statuses).

### Meteorological and air pollution data

Daily meteorological data were provided by the Meteorological Administration of each study city. These data included daily maximum, mean, and minimum temperatures (°C) and mean relative humidity (RH%). Daily air pollution data including particulate matter <2.5 µm in aerodynamic diameter (PM_2.5_, 24-h mean µg/m3) and ozone (O_3_, 8-h mean µg/m3) were obtained from municipal environmental monitoring sites in Chengdu, Zigong, Guangyuan, and Panzhihua.

### Statistical analysis

#### First stage analysis

The distributed lag non-linear models (DLNM) was developed by Gasparrini in 2010, a modelling framework that can simultaneously represent non-linear exposure-response dependencies and delayed effects [24]. A Poisson-distributed distributed lag non-linear model was used to evaluate the association between the extreme temperature and non-accidental mortality for each city in our study. In this study, the minimum mortality temperature (MMT) was used as the reference temperature, which corresponds to a minimum mortality percentile between the first and the 99th percentiles, was derived from the best linear unbiased prediction of the overall cumulative exposure-response association in each location [15]. We referred to the MMT as the optimum temperature, and used it as the reference for calculating the attributable risk.The model was as follows:

$$\begin{array}{rl} \text{Log[E(Yit)]} & =\alpha +\text{cb(Temp}{}_{\text{it}}\text{)}+\text{ns(RH,4)}\\ & \;+\text{ns(PM}{}_{2.5}\text{,4)}+\text{ns(O}{}_{3}\text{,4)}\\ & \;+\text{ns(Time}{}_{\text{i}}\text{, df*year)}+\text{DOW}, \end{array}$$

In this model, Yit represents the number of non-accidental deaths in city *i* on day *t*; cb(Tempit) is the cross-base matrix generated by DLNM with a maximum lag time of 25 days, which included a quadratic B spline with three internal knots placed at the 25th, 50th, and 75th percentiles of location specific temperature distributions, and the lag response curve with a natural cubic B spline with an intercept and three internal knots placed at equally spaced values in the log scale [15, 25]; ns(RH,4), ns(PM_2.5_,4), and ns(O_3_,4) represents natural cubic spline functions with four degrees of freedom; RH is the daily average relative humidity; ns(Time_i_, df*year) means spline functions with eight degrees of freedom per year to control for seasonal and long-term trends; DOW means the day of the week effect.

#### Second stage analysis

In the second stage, we used a multivariate meta-analysis to obtain summary estimates for the four cities [26]. A BLUP approach involved a trade-off between city-specific associations and second-stage pooled estimation, providing more precise estimates, especially in cities with small numbers of deaths [16]. We then calculated the number of attributable deaths and the proportion of attributable deaths during the present day and 25 lagged days according to previous studies [27]. Attribution fractions for cold (below MMT) and heat (above MMT) were calculated by deeming the MMT as the baseline reference. Based on the cutoff values of the 1% and 99% temperature percentiles and the MMT, we divided the temperature of each city into four levels, i.e., extreme cold (lower than the 1st percentile of the daily mean temperature), moderate cold (from the 1st percentile of the daily mean temperature to the MMT), moderate heat (from the MMT to the 99th percentile of the daily mean temperature), and extreme heat (greater than the 99th percentile of the daily mean temperature). Finally, the 95% empirical confidence intervals (eCIs) of attributable mortality were computed using Monte Carlo simulation [27]. Additionally, we also stratified the analysis by gender, age group, education level, and marital status through the above steps.

#### Sensitivity analysis

To test the stability of the model, we observed the exposure-response relationship by adjusting the maximum lag days (21/25 days), the df of the time trend (7–9), and we also controlled for ozone and fine particulate matter (PM_2.5_) in the sensitivity analysis.

We used R software 4.1.2 for data analysis. Specifically, the “dlnm” package [25] was used to estimate city-specific temperature-mortality associations, and the “mvmeta” package [28] was used for the meta-analysis.

## Results

Table 1 shows descriptive data for total non-accidental deaths, mean temperature, minimum temperature, maximum temperature, mean relative humidity, and air pollutants for four cities in the Sichuan Basin during the study period. The total number of deaths recorded in the four cities was 751,930. The total numbers of deaths in Chengdu, Zigong, Guangyuan, and Panzhihua were 530,228, 127,446, 68,061, and 26,195, respectively. The number of daily non-accidental deaths in each city varied widely, from 18 in Panzhihua to 242 in Chengdu. The daily mean temperature and daily mean relative humidity in the four cities were 18.3 °C (−1.6 °C–34.6 °C) and 72.4% (11.8%–100%), respectively. The mean concentrations of PM_2.5_ and O_3_ were 41.6 µg/m3 (3.8 µg/m3–300.8 µg/m3) and 85.4 µg/m3 (5.0 µg/m3–278.0 µg/m3), respectively.

**Table 1:** Descriptive statistics of general information for four cities in the Sichuan Basin, Southwest China

**Variables**	**Chengdu (2016–2021)**				**Zigong (2016–2021)**				**Guangyuan (2018–2021)**				**Panzhihua (2018–2021)**			

	**Mean ± SD**	**Max**	**Median**	**Min**	**Mean ± SD**	**Max**	**Median**	**Min**	**Mean ± SD**	**Max**	**Median**	**Min**	**Mean ± SD**	**Max**	**Median**	**Min**
Total death	241.9 ± 44.0	410.0	234.0	140	58.1 ± 12.5	130.0	57.0	25.0	46.6 ± 10.6	100.0	45.0	19.0	17.9 ± 4.8	40.0	18.0	4.0
Mean temperature (°C)	16.8 ± 7.4	30.5	17.1	−1.6	18.9 ± 7.4	34.6	19.1	0.8	16.3 ± 7.6	29.7	16.7	−1.0	21.6 ± 5.6	33.6	22.3	4.8
Minimum temperature (°C)	13.5 ± 7.3	26.6	14.3	−6.2	16.1 ± 6.8	30.1	16.4	−0.8	12.5 ± 7.5	25.5	12.8	−5.5	16.4 ± 5.8	28.5	17.5	2.6
Maximum temperature (°C)	21.4 ± 8.1	37.8	21.6	3.3	22.8 ± 8.3	40.9	23.2	3.1	21.4 ± 8.4	38.0	21.7	1.8	28.5 ± 5.4	40.5	28.8	6.3
Relative humidity (%)	79.7 ± 9.4	99.0	80.6	36.0	78.6 ± 12.1	100.0	80.0	34.0	70.1 ± 14.6	99.0	71.0	14.0	54.2 ± 20.0	98.3	57.3	11.8
PM_2.5_ (µg/m3)	47.4 ± 33.0	256.5	38.2	3.8	54.0 ± 37.4	300.8	42.8	7.8	26.5 ± 17.2	132.0	22.0	5.5	29.4 ± 12.7	119.1	27.7	7.3
O_3_ (µg/m3)	94.2 ± 47.2	278.0	84.1	10.3	82.7 ± 38.5	250.6	75.2	13.0	72.9 ± 31.6	193.0	71.0	5.0	88.9 ± 30.4	183.1	84.4	15.3

Table 2 shows the overall and heat and cold estimated attribution fractions for the four cities. Overall, the MMP was 80% (25.1 °C), and 10.16% of non-accidental mortality was attributed to cold and heat. The MMP distribution was around 80% in all cities, and the MMT distribution was between 20 °C and 30 °C. The majority of temperature-related non-accidental deaths were attributed to cold, accounting for 9.10% (95% eCI: 5.50%, 12.19%), and heat effects accounted for only 1.06% (95% eCI: 0.76%, 1.33%). This difference was caused by a higher minimum mortality percentile, with most daily mean temperatures being below the MMT.

**Table 2:** The attributable fraction of non-accidental mortality associated with cold and heat in southwest China cities

**City**	**MMP**	**MMT**	**Total (%)**	**Cold (%)**	**Heat (%)**
Chengdu	81	24.6	10.86(5.98–15.82)	9.81(4.91–14.11)	1.05(0.68–1.42)
Zigong	75	25.0	7.63(3.58–10.99)	6.68(2.77–10.33)	0.95(0.27–1.57)
Guangyuan	78	23.6	9.81(4.54–14.06)	8.52(4.11–12.70)	1.29(0.82–1.76)
Panzhihua	84	27.2	9.05(4.45–13.25)	8.07(3.47–12.12)	0.98(0.59–1.32)
Total	80	25.1	10.16(6.59–13.62)	9.10(5.50–12.19)	1.06(0.76–1.33)

MMP: Minimum Mortality Percentile; MMT: Minimum Mortality Temperature

**Fig. 2:**
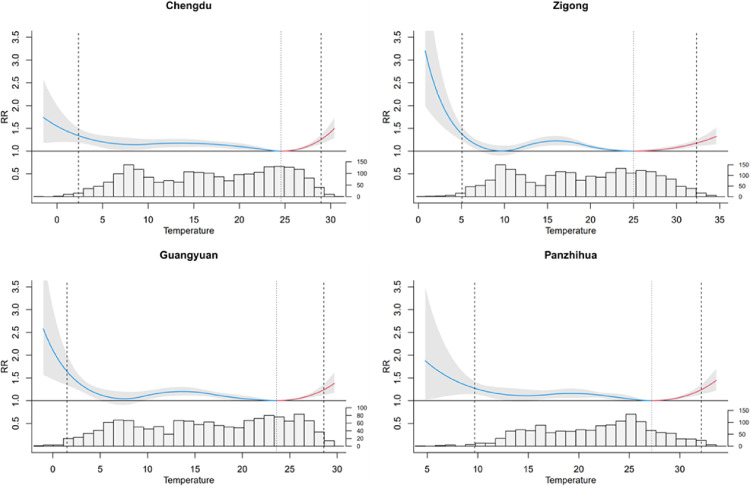
Overall cumulative exposure-response correlations for the four cities. Exposure-response associations are presented as the best linear unbiased prediction (95% empirical CI, shaded in gray) for the four cities and the associated temperature distribution. The gray dashed line indicates the lowest mortality temperature, and the short black lines denote the 1st and 99th percentiles. RR = relative risk.

**Fig. 3:**
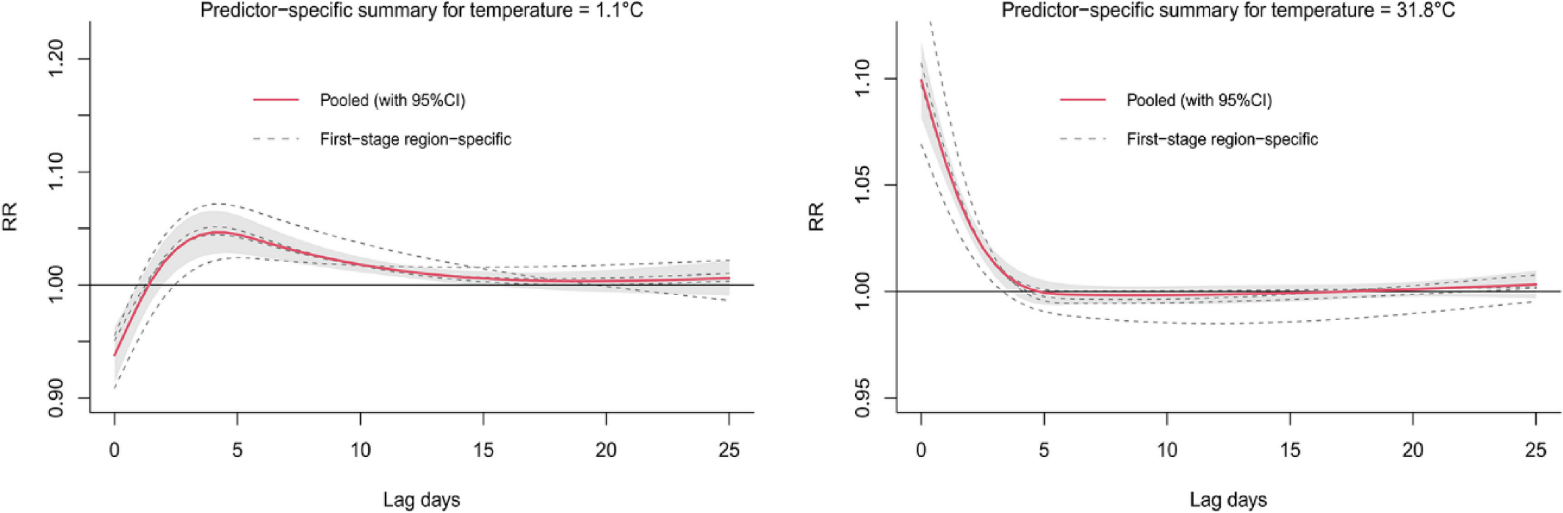
Overall lag structure in effects of extreme temperatures on non-accidental mortality in studied cities. Effects were defined as the relative risks at 31.8 °C (that is, the mean of the 99^th^ centile of temperature distributions) and 1.1 °C (that is, the mean of the 1^st^ centile of temperature distributions) compared with the estimated minimum mortality temperature.

Figure 2 shows the best linear unbiased predicted (BLUP) estimates of the exposure–response relationships between temperature and non-accidental mortality in the four cities, with corresponding MMT and the cutoffs to define extreme and moderate temperatures. The temperature-mortality association curve was described by a U or L shape. The RRs of extreme heat and cold were higher; the cold effect lasts longer, and most of the daily mean temperatures were distributed below the MMT. Figure 3 shows the effects of the extreme heat (31.8 °C) and extreme cold (1.1 °C) on non-accidental mortality at lags of 0–25 days. The effect of extreme heat is immediate and disappears after 3–4 days. The effect of extreme cold lasted for a long lag period.

**Fig. 4:**
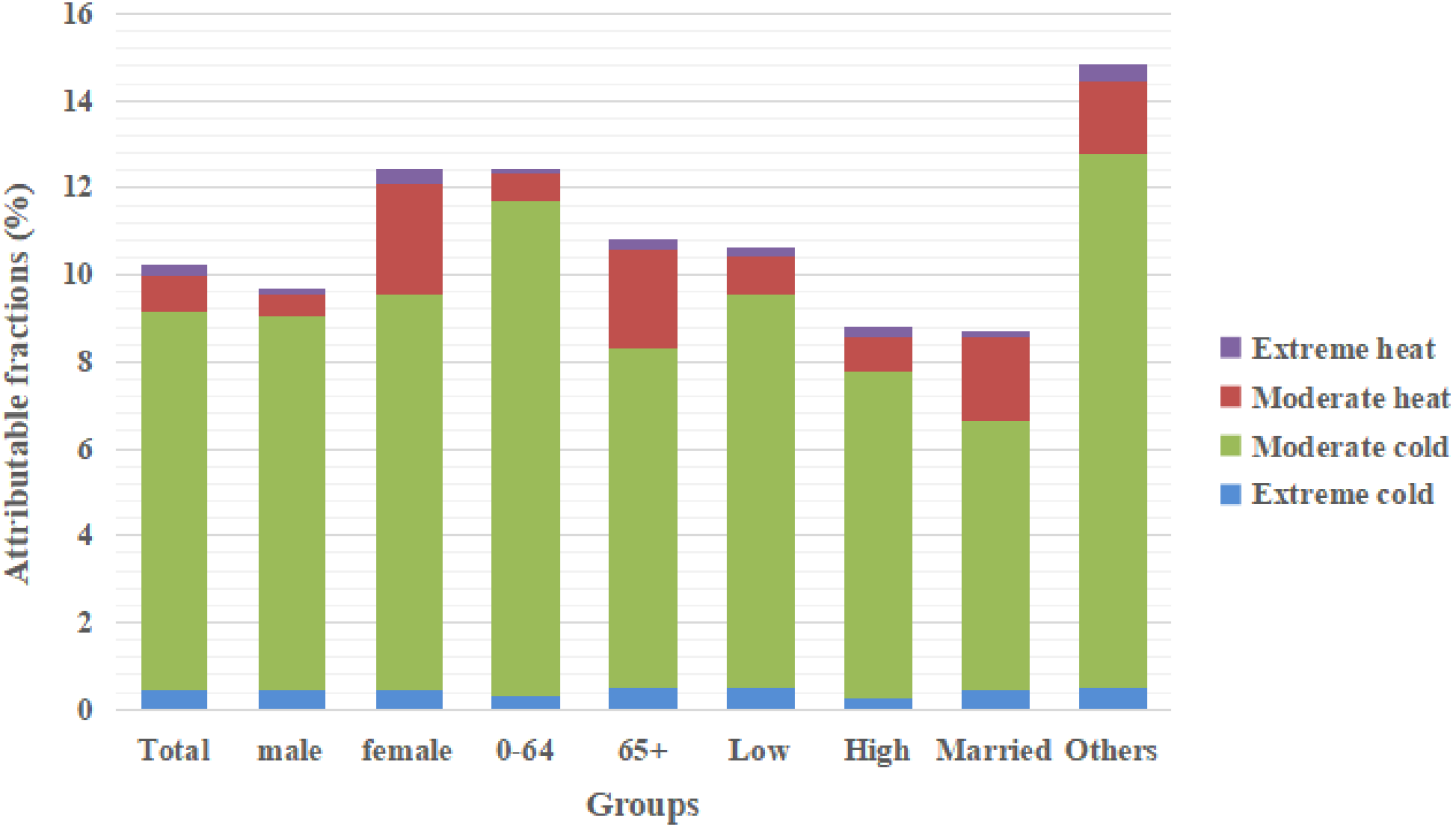
Attribution fractions for non-accidental mortality due to heat and cold, grouped by different categories. Low: illiterate and primary; high: junior high school and above. Others: widowed, divorced, and never married.

Figure 4 presents the attributable fractions of low and high temperatures for non-accidental mortality in subgroups by sex, age, education level, and marital status. Overall, AF was significantly higher for moderate temperatures than for extreme temperatures. The greatest proportion of non-accidental deaths were attributable to moderate cold (from 6.23% to 12.30%), with only a small proportion of non-accidental mortality attributable to extreme cold (0.24%–0.48%) or extreme heat (0.10%–0.37%). Those over 65 years, females, and those with a low education level or alternative marriage status were more sensitive to extreme temperatures. The mortality burden attributable to non-optimal temperatures was higher among those under 65 years old, females, and those with low education level or alternative marriage status, with overall AF of 12.42% (95% eCI: −1.85%, 21.90%), 12.37% (95% eCI: 6.71%, 17.43%), 10.54% (95% eCI: 6.56%, 11.11%), and 14.74% (95% eCI: 9.56%, 18.97%), respectively.

## Discussion

Our study assessed the associations between low and high temperatures and non-accidental mortality using more than 0.7 million deaths from four cities in the Sichuan Basin of southwest China, and we further estimated the mortality burden attributable to heat and cold. The results showed that both heat and cold significantly increased the mortality risk. Overall, the MMP was 80%, and 10.16% of non-accidental deaths could be attributed to non-optimal temperatures. The majority of temperature-related non-accidental deaths were caused by cold temperature at 9.10%, with heat effect accounting for only 1.06%. We observed that low temperature had a greater effect on mortality and lasted longer, while the heat effect was immediate, consistent with previous studies [29, 30]. Stratified analysis by age, sex, education level, and marital status showed that those over 65 years of age, females, and those with low education level or alternative marriage status were more sensitive to extreme temperatures, while the mortality burden attributable to non-optimal temperatures was higher among those under the 65 years old, females, and those with a low education level or alternative marriage status.

Many studies have found that the effects of temperature on human health were generally characterized by “U,” “V,” and “J” shapes [31, 32]. Our study found that temperature was associated with mortality in a “U” or “J” shape, with both hot and cold increasing the risk of death, and low temperature having a higher effect than high temperature. This is consistent with previous findings [15, 16, 33]. For example, Chen et al. found that the attributable percentages of total non-accidental mortality, cardiovascular disease, and respiratory disease mortality caused by non-optimal temperature in 272 cities in China were 14.33%, 17.48%, and 10.57%, respectively. The mortality risk of extreme cold temperature lasted for more than 14 days, whereas the risk of extreme hot temperature appeared immediately [16]. Gasparrini et al. also found that the total proportion of deaths caused by heat and cold was 7.71% in 384 locations, and more temperature-attributable deaths were caused by cold (7.29%) than by heat (0.42%) [15]. A study by Scovronick et al. found that 3.4% of deaths (∼290,000) in South Africa were attributable to non-optimum temperatures during the study period, with heat effects occurred immediately after exposure and diminished rapidly, while cold effects were delayed and persistent [33].

Temperatures in the Sichuan Basin are higher than in other regions at the same latitude, with extreme high temperatures in the southeastern part of the basin often exceeding 40 °C. Our results showed that 10.16% of non-accidental deaths were attributable to non-optimal temperatures, similar to the findings of Gasparrini [15] and Ma [10], lower than those of Chen [16] and Zhang [34], and higher than those of Scovronick [33] and Cao et al. [35] The different results may be related to the different study locations, socioeconomic characteristics, life patterns, medical conditions and demographic characteristics. According to Bannister’s study, under the CMIP5 high-emission future climate change scenario, the temperature in the Sichuan Basin is expected to increase by about 4 °C by 2100, and more frequent extreme temperature events could have adverse effects on population health [21]. Therefore, it is important to develop adaptation plans to reduce the adverse effects of climate change.

Previous studies have confirmed that individual social factors (e.g., age, sex, and marital status) can modify the effect of temperature on mortality [36, 37]. Our study found that the elderly over 65 years, females, and those with a low education level or alternative marriage status were more sensitive to extreme temperatures. This is consistent with some previous studies [33, 38, 39], but inconsistent results were observed in other studies [9]. A study in South Africa found that people over the age of 65 were more sensitive to extreme temperatures [33]. Physiological studies have shown that most elderly individuals have comorbidities (co-morbidities) that make them more sensitive to extreme temperatures or temperature changes than younger adults. Moreover, elderly have weaker neurothermoregulatory mechanisms and reduced thermoregulatory capacity and may not be able to maintain their core temperature at safe levels, and prolonged exposure to temperature extremes may lead to associated diseases or other fatal events [40–42]. We found that women were more susceptible to the effects of heat and cold than men. Previous studies have yielded mixed results, with some studies finding a higher risk of temperature-related mortality in women than in men [16, 36, 43]. However, some studies also reported a greater sensitivity to extreme temperatures in men [9]. Previous studies found that people with low or no education were at higher risk of heat stroke [37, 44], consistent with our results. Education may be an indicator of socioeconomic status, and this may be associated with poor baseline status, limited health care coverage, and associated housing conditions [38]. Only a few studies have analyzed the modifying effect of marital status on temperature in the past. Son et al. found a higher risk of death due to heat and cold in widows [37]. Our results showed that individuals with alternative marriage status were more susceptible to extreme temperatures.

In cold conditions, body core temperature drops, resulting in the depletion of physical reserves of the heart, liver, muscles, breathing, and heartbeat, which may manifest as shivering, respiratory depression, cardiac arrhythmias, and impaired mental functions, causing vessel spasm development and inefficient circulation, even progressing to cardiac arrest or coma [45–47]. One study found that hypothermia deaths were twice as frequent as hyperthermia deaths [48].

Under conditions of high temperature, the thermoregulatory capacity of the body may be exceeded, leading to illness due to overheating. If the resultant body water deficit is not adequately replenished, it can lead to dehydration. This may lead to heat stroke and increased cardiovascular strain, and even death [49, 50]. In addition, high internal body temperature (39–40 °C), increased ischemia, and oxidative stress after blood redistribution can lead to cell, tissue, or organ damage, with organs such as the brain, heart, kidneys and lungs being at greater risk [4].

Our study suggest that the local government should pay more attention to vulnerable people and take measures to reduce adverse effects in extreme climates. On the one hand, various forms of targeted activities for publicity and education ought to be carried out. Provide scientific knowledge on the correct response and self-rescue in the event of extreme weather to enhance the public’s awareness of self-protection. On the other hand, local government should actively carry out assessment and investigation of the impact of extreme weather on the health of the population, improve and optimize the early warning system of heat and cold waves health risks.

In order to ensure the stability of the study results, we conducted sensitivity analyses. The lag days were chosen to be 21 and 25 days; the time trend degrees of freedom was from 7 to 9, and the mean relative humidity and pollutants ranged from 3 to 5 (Table S1). The AF calculated according to different df were similar. Therefore, the results calculated by the model were reliable.

To our knowledge, this is the first study to explore the association between non-appropriate temperatures and mortality in a multi-city area of the Sichuan Basin in southwest China. Second, the mortality data were derived from the PDIRMS and were therefore authentic and reliable. In addition, we conducted subgroup analyses, which were more comprehensive and included age, sex, education level, and marital status, to better reveal how susceptible populations are affected by non-optimal temperatures. However, our study also has some limitations. Since ecological studies have inherent limitations, we could not obtain individual exposure data. In addition, our study included four cities in the central, northern, and southern parts of the Sichuan Basin in southwest China, while other cities were not included. Therefore, the results may not generalize to other regions.

## Conclusions

Our study indicates that a significant association between non-optimal temperature and non-accidental mortality. Most temperature-related deaths were caused by low temperatures, with moderately high and low temperatures representing the majority of the mortality burden. Those under 65 years old, females, and those with a low educational level or alternative marriage status had the highest attributable burden. Our findings may be helpful to policymakers at local levels in developing adaptation plans.

## Abbreviations

PM2.5: particulate matter <2.5 µm in aerodynamic diameter
O3-8h: daily 8-hour mean concentrations of ozone
RH: relative humidity
MMT: minimum mortality temperature
MMP: minimum mortality percentile
Afs: attributable fractions
DLNM: distributed-lag non-linear models
SD: standard deviation
RR: relative risk
CI: confidence interval

## Supplementary information

**Additional file 1:** **Table S1** Computed on the attributable fraction (%) to temperature (total, heat, and cold components), by varying lag, df, and controlling air pollution.

## Declarations

### Availability of data and material

The datasets used in this study are available from the corresponding author upon reasonable request.
