# Supplementary material for: Association between temperature and mortality: a multi-city time series study in Sichuan Basin, southwest China
Source: Environ Health Prev Med. 2024 Jan 12;29:1. doi: 10.1265/ehpm.23-00118 (PMC10788187; doi:10.1265/ehpm.23-00118)
Supplement: Supplementary file 1 — Additional file 1: Table S1 Computed on the attributable fraction (%) to temperature (total, heat, and cold components), by varying lag, df, and controlling air pollution. [file ehpm-29-001-s001.docx]

Table S1 Computed on the attributable fraction (%) to temperature (total,heat, and cold components), by varying lag, df, and controlling air pollution

| Value | MMP | Overall (%) | Heat (%) | Cold (%) |
| --- | --- | --- | --- | --- |
| Lag days: 15 (4cities) | 79 | 10.29(4.00-15.66) | 1.03(0.46-1.58) | 9.26(2.94-14.86) |
| Lag days: 21(4cities) | 80 | 10.09(6.79-13.33) | 0.97(0.70-1.20) | 9.12(5.84-12.29) |
| Lag days: 25(4cities) | 80 | 10.16(6.59-13.62) | 1.06(0.76-1.33) | 9.10(5.50-12.19) |
| Time Trends DF: 7 | 87 | 9.35(6.17-12.30) | 0.83(0.25-1.32) | 8.52(5.31-11.60) |
| Time Trends DF: 9 | 77 | 10.99(4.31-16.28) | 2.06(-0.31-3.67) | 8.93(2.91-14.14) |
| RH:3 | 80 | 10.22(6.57-13.49) | 1.06(0.78-1.33) | 9.15(5.45-12.55) |
| RH:5 | 80 | 10.12(6.36-13.33) | 1.06(0.79-1.33) | 9.07(5.53-12.30) |
| Without PM_2.5_+ O_3_ | 79 | 10.75(7.54-14.00) | 1.14(0.84-1.41) | 9.61(6.08-12.68) |
